# Iodine Nutrition in Children ≤2 years of Age in Norway

**DOI:** 10.1016/j.tjnut.2023.09.013

**Published:** 2023-09-22

**Authors:** Tonje E. Aarsland, Beate S. Solvik, Kjersti S. Bakken, Synnøve Næss Sleire, Siri Kaldenbach, Mads N. Holten-Andersen, Kristina R. Nermo, Ingunn T. Fauskerud, Thobias H. Østvedt, Solfrid Lohne, Elin L.F. Gjengedal, Tor A. Strand

**Affiliations:** 1 Center of International Health, Department of Global Public Health and Primary Care, University of Bergen, Bergen, Norway; 2 Women’s Clinic at Lillehammer Hospital, Innlandet Hospital Trust, Lillehammer, Norway; 3 Seafood, Nutrition and Environmental State, Institute of Marine Research, Bergen, Norway; 4 Department of Paediatric and Adolescent Medicine, Innlandet Hospital Trust, Lillehammer, Norway; 5 Clinical Medicine, Faculty of Medicine, University of Oslo, Oslo, Norway; 6 Department of Microbiology, Innlandet Hospital Trust, Lillehammer, Norway; 7 Faculty of Environmental Sciences and Natural Resource Management, Norwegian University of Life Sciences, Ås, Norway; 8 Department of Research, Innlandet Hospital Trust, Lillehammer, Norway

**Keywords:** urinary iodine concentration, breast milk iodine concentration, iodine status, iodine intake, 24-h dietary recall, Multiple Source Method, females of childbearing age, lactating females, infants, toddlers

## Abstract

**Background:**

As a component of the thyroid hormones (THs), iodine is vital for normal neurodevelopment during early life. However, both deficient and excess iodine may affect TH production, and data on iodine status in young children are scarce.

**Objectives:**

To describe iodine nutrition (iodine status and intake) in children ≤2 y of age in Innlandet County (Norway) and to describe the associations with maternal iodine nutrition.

**Methods:**

A cross-sectional study was performed in a representative sample of mother–child pairs selected from 30 municipalities from November 2020 until October 2021. Iodine status [child urinary iodine concentration (UIC), maternal UIC, and breast milk iodine concentration (BMIC)] was measured. Child’s iodine intake was estimated using 2 24-h dietary recalls (24-HR) and a food frequency questionnaire. The Multiple Source Method was used to estimate the usual iodine intake distributions from the 24-HR assessments.

**Results:**

The median UIC in 333 children was 145 μg/L, indicating adequate iodine status according to the WHO cutoff (100 μg/L). The median usual iodine intake was 83 μg/d. Furthermore, 35% had suboptimal usual iodine intakes [below the proposed Estimated average requirement (72 μg/d)], whereas <1% had excessive usual iodine intakes [above the Upper intake level (200 μg/d)]. There was a positive correlation between children’s iodine intake and BMIC (Spearman rank correlation coefficient *r* = 0.67, *P* < 0.001), and between children’s UIC and BMIC (*r* = 0.43, *P* < 0.001), maternal UIC (*r* = 0.23, *P* = 0.001), and maternal iodine intake (*r* = 0.20, *P* = 0.004).

**Conclusion:**

Despite a median UIC above the cutoff for iodine sufficiency, more than a third of the children had suboptimal usual iodine intakes. Our findings suggest that many children will benefit from iodine fortification and that risk of iodine excess in this age group is low.

## Introduction

Several recent studies in Norway and elsewhere have documented poor iodine intake and iodine deficiency (ID) in females of childbearing age, including pregnant and lactating females [[1], [2], [3], [4], [5], [6], [7], [8], [9]. In addition, estimates have proposed that ≤50% of newborns in Europe are exposed to ID [10]. These findings have raised concerns regarding the iodine status of infants and young children, given the importance of thyroid hormones (THs) in the development of the central nervous system [11].

Iodine is an essential micronutrient required in the production of the THs, triiodothyronine and thyroxine [12]. From the fetal stage into postnatal life, THs are crucial for neural and cognitive development and regulate many vital processes in the body [13]. In early life, the production rate of THs per kg bodyweight is particularly high as the body undergoes rapid growth, leading to an increased turnover of intrathyroidal iodine stores [14]. Hence, infancy and early childhood are particularly critical periods for ID. Suboptimal iodine intakes in this period may lead to iodine depletion, diminish TH synthesis, and thereby affect physical, neurological, and cognitive development [15]. During the first 4–6 mo of life, adequate iodine intake for TH production must be provided through human breast milk and/or formula. The iodine concentration in breast milk reflects maternal iodine intake in the past hours before breastfeeding, and thus, the iodine intake of exclusively breastfed infants relies entirely on maternal iodine status and intake [16]. Furthermore, with the introduction of complementary foods, iodine is also obtained from iodine-rich food sources, such as enriched infant porridge, lean fish, eggs, and cow’s milk [17,18].

Globally, there has been a remarkable improvement of iodine intake over the past decades, mainly because of salt iodization programs. Yet, ID remains a threat in countries where efficient salt iodization programs are not established, such as Norway [19]. As part of a strategy to improve iodine intake in Norway, the government decided in 2023 to increase the permitted level of iodine in table salt and salt used in industry bread and bakery products from 5 μg/g to 20 μg/g [20]. However, the food industry has not yet implemented the increase in their production, and so far, they have not received any explicit encouragement from the health authorities to do so. Because both ID and iodine excess may impair TH synthesis [21], it is important to monitor the population’s iodine status, particularly vulnerable population groups such as young children and lactating females. Several studies have documented ID among lactating females in Norway [[3], [4], [5],^22^]; however, studies reporting on iodine nutrition in early childhood are rather scarce. In most previous studies, iodine intake has been estimated in older children or breastfed children have been excluded from estimates of iodine intake because of limited knowledge of consumption volumes.

On the basis of the above, the aim of this study was to examine iodine nutrition (iodine status and intake) in a representative sample of children ≤2 y of age in Innlandet County (Norway). Furthermore, we aimed to assess the associations between the iodine nutrition of children and their mothers, and to describe the main dietary sources of iodine in young children.

## Methods

### Study design and study population

In this cross-sectional study, mother–child pairs from public health care centers were recruited from 30 randomly selected municipalities in Innlandet County in Norway from November 2020 until October 2021. During consultations at the health care centers, nurses approached and recruited females who could communicate in Norwegian and had a child between 0 and 2 y of age with no known ongoing, congenital, or chronical illness. The selection and recruitment process has been described in more detail previously [23].

### Collection of urine samples and human breast milk

As recommended by the WHO/UNICEF/ICCIDD, iodine status was measured by urinary iodine concentration (UIC) in spot urine samples of children and their mothers [12]. Iodine status in lactating mothers was also measured by breast milk iodine concentration (BMIC). Females who agreed to participate were instructed to collect a urine sample from herself and her child, and a breast milk sample (if applicable). Oral and written instructions and a bag of equipment were provided by trained nurses at the health care centers.

A spot urine sample from the child (1–5 mL) was collected using a urine collection pad placed in the diaper (Sterisets Urine collection packs, Sterisets International BV), checking the diaper every 10 min to retrieve the urine before possible feces contamination. If the pad was soiled, then a second attempt was made using a clean pad. Once the pad was wet with urine, it was placed with the wet side up and the urine was transferred into a sterile urine specimen container using a syringe. In case of difficulty extracting the urine using the syringe, an alternative method was to open the pad and squeeze the urine from the wet cotton directly into the container. A reliability study showed that compared with standard methods of collecting urine for measuring UIC, the diaper-pad collection method did not substantially affect the reliability of the measurements [24].

A spot urine sample from the mother was collected using a 100 mL Vacuette® urine beaker (Greiner Bio-one) and a 9.5 mL Vacuette® Urine tube (Greiner Bio-One). Lactating mothers also provided a spot breast milk sample (20 mL, or any volume below she was able to provide) by manual or mechanic expression into a 50 mL polypropylene centrifuge tube (Sarstedt). For compliance and convenience, the mothers could sample the urine and breast milk at any given time point during the day. The samples were collected at home, marked with unique ID numbers, kept in the fridge, and delivered to the health care center within 1 wk after sampling. All samples were kept in the fridge (4°C) until picked up by the study team.

The samples were aliquoted into vials (5 and 10 mL for breast milk and 2 or 5 mL for urine) before long-time storage at −80°C. All samples were thawed at 4°C overnight or at room temperature the same day. To homogenize the samples, they were incubated at 38°C for 1 min in an ultrasonic bath. Eppendorf Xplorer® 0.2–5 mL (VWR 613-1408) was used to transfer 1 mL sample to 10 mL falcon tubes (SARSTEDT 62.554.502) using Eppedorf epT.I.P.S.® (VWR 613-6934). The falcon tubes were measured (Sartorius MSE125P-100-DU) before and after sampling for optimal weight measurement. Samples were frozen at −80°C until they were transported to the laboratory (Faculty of Environmental Sciences and Natural Resource Management at the Norwegian University of Life Sciences, Norway) for analyses.

### Determination of iodine in urine and human breast milk

The 1.00-mL aliquot of breast milk was diluted to 10.0 mL with an alkaline solution (BENT), containing 4% [weight (*w)*/volume (*V)*] 1-**b**utanol, 0.1% (*w/V*) H_4_**E**DTA, 5% (*w/V*) **N**H_4_OH, and 0.1% (*w/V*) **T**riton™ X-100. Method blank samples and samples of standard reference material (SRM) were prepared following the same procedures. The samples were analyzed for iodine by means of an Agilent 8800 ICP-QQQ (Triple Quadruple Inductively Coupled Plasma Mass Spectrometer, Agilent Technologies) using oxygen as a reaction gas. Iodine was determined at mass 127. ^129^I was used as an internal standard for ^127^I. The quantification of iodine in spot urine followed the same procedure, except that the urine was thawed, but not heated before the alkaline dilution, and the concentration of NH_4_OH in BENT was decreased to 2% to limit precipitation of struvite (MgNH_4_PO_4_⋅6H_2_O) in urine. Reagents of analytical grade or better and deionized water (>18 MΩ) were used throughout.

The limits of detection (LOD) and limits of quantification (LOQ) were calculated by multiplying the standard deviation of the method blank samples (*n* = 18) by 3 and 10, respectively. The obtained LOD and LOQ for iodine were 0.20 and 0.79 μg/L, respectively. To ensure methodological traceability and to check for accuracy, SRM were analyzed concurrently with the sample matrices. Accuracy in the determination of iodine in breast milk was checked by analysis of the European Reference Material ERM®-BD150 Skimmed milk powders. Considering urine, Seronorm^TM^ Trace Elements Urine L-1 and Seronorm^TM^ Trace Elements Urine L-2 (Sero AS) were analyzed; each with value assignment established in accordance with International Organization for Standardization 17511. Allowing for experimental error, that is, a coverage factor *k* = 2, corresponding to a level of confidence ∼95%, our results were within the recommended values issued. Intermediate precision (within-laboratory reproducibility) in the analysis of urine for iodine was 3.9% (*n* = 11).

### Collection of dietary data and estimation of iodine intake

To estimate usual iodine intake, short-term measurements such as 24-h dietary recalls (24-HRs) are the preferred method [25]. Thus, the main method for the collection of dietary data in this study was 2 24-HRs per child. The children’s mothers reported the dietary data by phone to 1 of the 3 certified dietitians in the study team. To capture iodine-rich food sources that are irregularly consumed, dietary data were also obtained using an electronic food frequency questionnaire (FFQ) [17,18].

We aimed to conduct 2 24-HRs for each child, with different time intervals (3 d–3 wk) and on different days of the week. Non-responding females were contacted and reminded a maximum of 3 times. The structure of the interview varied with the complexity of the child’s diet; however, all interviews were initiated with questions about the intake of breast milk and/or formula. If formula was consumed, then the type of formula and the total volume ingested were reported. Iodine intake from the formula was calculated by multiplying the iodine concentration of the given formula (retrieved from the product declaration) with the reported consumption volume. The volume of ingested breast milk was only reported if the milk was bottle-fed. For breastfed children, the volume consumed was derived from recently published global age-specific breast milk intake estimates by Rios-Leyvras et al. [26]. Furthermore, to calculate the iodine contribution from breast milk, the consumption volume was multiplied by the BMIC of the corresponding mother, if measured. If the BMIC was not measured, then the iodine concentration from the Norwegian Food Composition Table of 70 μg**/**L was used [27]. If intake of other kinds of milk in addition to breast milk was reported (formula, cow’s milk, or plant-based milk replacements), then the volume of consumed breast milk was reduced by the corresponding volume for these. For instance, the mean daily intake of breast milk for an 8-mo-old infant is 694 mL/d [26]. If an infant of this age received 200 mL of formula in addition to breast milk, 200 mL was subtracted from 694 mL. If the volume of other kinds of milk than breast milk exceeded the age-specific estimate, then the volume of breast milk consumption was set to 0 (unless the breast milk was bottle-fed). For children who were weaned or received complementary foods, the mother was further asked to describe as freely as possible what her child consumed the day before the call, from when the child woke up and the following 24 h. Next, the mother was asked to provide details on portion sizes, ingredients, and preparation methods of home-cooked meals. Finally, the interviewer ensured that nothing was missed using a list of easily forgotten foods (for example, butter/oil in cooking, snacks between meals, dietary supplements, and foods eaten while outside the home). The Norwegian dietary estimation tool “Kostholdsplanleggeren” [28] was used to compute total daily intakes of iodine and energy. This tool multiplies the consumption amount of each food item with the concentration of the corresponding nutrient registered in the Norwegian Food Composition Table [27]. For items not found within this system, we chose similar items when appropriate, or obtained nutritional values from the producers of the food items.

Two different semi-quantitative FFQs were applied, one for children <6 mo of age and the other for older children. Depending on the child’s age, one of these was answered online by either of the child’s parents. The FFQs were originally developed for the third national dietary survey among infants in Norway conducted in 2018 and 2019 among 6- and 12-mo-old infants, respectively (“Spedkost 3”) [17,18]. Respondents were asked to report the child**’**s habitual diet with the last 2 wk in mind. Both FFQs included questions about breastfeeding, formula, and dietary supplements. For some foods, picture series were included to help aid in estimating portion sizes. Frequencies of intake varied with the food item in question and ranged from never/seldom to ≥5 times per wk or per mo. The food composition database and calculation system “Kostberegningssystem” (KBS, version AE-18) from the University of Oslo was used to calculate energy and nutrient intake. Non-responders were sent a maximum of 3 reminders (by text message) to answer.

Maternal iodine intake was also assessed using 2 24-HRs. These data have been described earlier [23] and are not further discussed in this paper, apart from its correlation with children’s iodine nutrition.

### Definitions of iodine status, iodine intake, and feeding practice

In children, we used the cutoff of a median UIC >100 μg**/**L for iodine sufficiency, which is the proposed cutoff from WHO for children <2 y of age [29]. The same UIC cutoff was applied for maternal iodine sufficiency [29]. Furthermore, for assessment of dietary iodine intake, an adequate intake was defined as an intake above or equal to the Adequate intake (AI) from the Nordic Nutrition Recommendations (NNR) 2023 of 90 μg/d for infants <12 mo of age and 100 μg/d for children ≥12 mo of age [30]. The NNR has not defined an Upper intake level (UL) or Average requirement (AR) of iodine for young children. Thus, we used the UL from the European Food Safety Authority of 200 μg/d for children 1–3 y of age to define excessive iodine intake [31]. Furthermore, we used the proposed Estimated average requirement (EAR) of 72 μg/d from a Swiss dose-response crossover balance study [32] to define suboptimal iodine intake.

The children were divided into the following 4 groups of feeding practice: breastfed (exclusively, or in combination with complementary foods, no use of formula); formula-fed (exclusively, or in combination with complementary foods, no breast milk); mixed milk-fed (breast milk and formula, with or without complementary foods); and weaned (only solid foods, no breast milk or formula). The current paper focuses on iodine nutrition in 3 age subgroups: <6, 6–11.9, and ≥12 mo.

### Statistics

The estimated sample size was based on the absolute precision of the estimated proportion with low iodine intake or deficiency. The calculated sample size for the project ensured a margin of error of 4% and a 95% confidence interval.

The proportion of children with usual iodine intakes below the AI, below the EAR (suboptimal intake), and above the UL (excessive intake) were estimated for all children and for the 3 age groups separately. Data processing and analyses was performed using STATA/SE 16.1 (StataCorp). To estimate the intake distributions from the 24-HR assessments, the Multiple Source Method (MSM) was used through the online interface accessible at https://msm.dife.de. The MSM is a statistical method that enables the estimation of usual dietary intake from repeated short-term measurements. The FFQ data were not used in the MSM analyses because the inclusion of such data is found to have minimal impact on the estimates of usual intakes from 24-HRs [25]. The results were calculated as μg/L iodine (UIC and BMIC), and μg/d (iodine intake), and were expressed as median (P25 and P75) because the data were skewed. We calculated the Spearman’s rank correlation coefficient *r* between children’s UIC and iodine intake and maternal variables of iodine nutrition (iodine intake, BMIC, and UIC). The strength of the correlation was considered poor if *r* < 0.20, moderate if 0.20–0.49, and strong if ≥0.50 [33]. The non-parametric Kruskal–Wallis test was used to assess whether the UIC and iodine intake differed across different child age groups (<6, 6–11.9, and ≥12 mo of age) and feeding practices (breastfed, formula-fed, mixed milk-fed, and weaned). A *P* value <0.05 was considered statistically significant.

### Ethics

Written informed consent was given by the mothers on behalf of their children. The study was approved by the Regional Committee for Medical and Health Research Ethics (2018/1230/REC South East).

## Results

**FIGURE 1:**
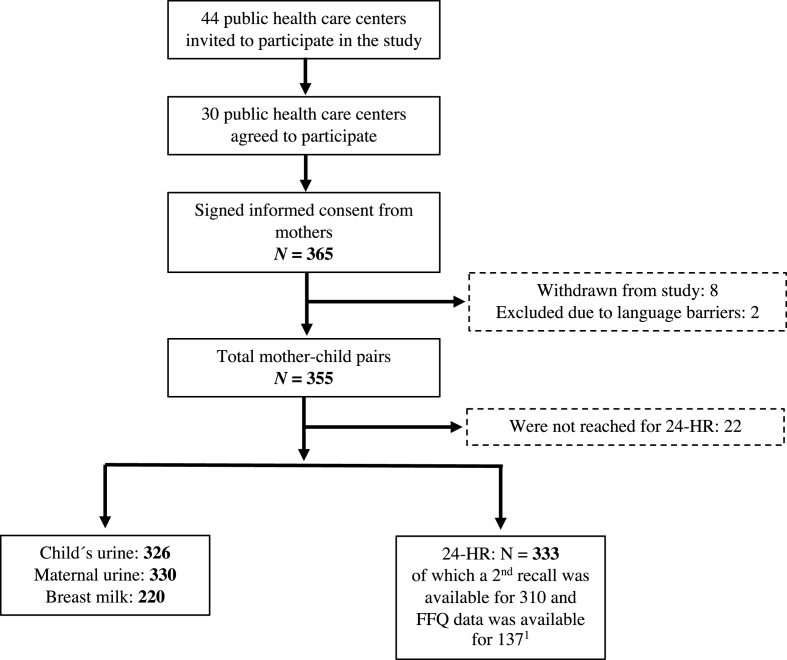
Flowchart of the study population and the collection of urine, breast milk, and dietary data (24-HRs and FFQ). 24-HR, 24-h dietary recall; FFQ, food frequency questionnaire. ^1^FFQ data were available for 251 children, but calculations of energy and iodine intakes were only computed for children ≥6 mo of age.

A total of 333 children were included in the estimations of iodine intake, of which urine samples for analyzation of iodine were available from 326. An overview of the study population and the collected data is given in Figure 1. More characteristics of the study population are given in Table 1. At the time point of the first 24-HR, the mean (SD) age of the children was 8 (6.0) mo. Furthermore, 3% had never been breastfed, 29% were previously breastfed, and 68% were still breastfed.

**TABLE 1:** Characteristics of mother–child pairs enrolled in the study

Characteristic	*n*	*n* (%) or mean (SD)
Child		
Age, in mo	333	
All children		8 (6.0)
<6		132 (39.7)
6–11.9		117 (35.1)
≥12		84 (25.2)
Sex, boy	328	58 (48.2)
Feeding practice	333	
Breastfed		196 (58.9)
Formula-fed		65 (19.5)
Mixed milk-fed		30 (9.0)
Weaned		42 (12.6)
Mother		
Maternal age, (y)	295	31.0 (4.4)
Maternal BMI (kg/m^2^)	288	
<18.5 (underweight)		1 (0.3)
18.5–24.9 (normal weight)		151 (52.4)
25–29.9 (overweight)		81 (28.1)
>30 (obese)		55 (19.1)
Marital status	295	
Single/other		10 (3.4)
Cohabitant		195 (66.1)
Married		90 (30.5)
Maternal education level	295	
<12 y		3 (1.0)
12 y		71 (24.1)
1–4 y college/university		161 (54.6)
>4 y college/university		60 (20.3)
Maternal country of birth, Norway	295	261 (88.5)
Maternal smoking status	295	
Daily		7 (2.4)
Previous smoker		25 (8.5)
No		263 (89.2)
Use of snuff	295	
Daily		22 (7.5)
Sometimes		4 (1.4)
Previous		21 (7.1)
No		248 (84.1)
Previous or current thyroid disease	295	15 (4.6)
Hyperthyroidism		6 (2.0)
Hypothyroidism		9 (3.1)
Current use of thyroid medication		8 (2.7)

### Children’s iodine nutrition

Data on children’s UIC and estimated iodine intake by the 24-HRs and FFQ, stratified by the 3 age groups (<6, 6–11.9, and ≥12 mo), are provided in Table 2. The iodine sources and their contribution (in %) to the iodine intake are presented in Table 3. None of the children were reported to consume iodine-containing supplements.

**TABLE 2:** UIC among the children and intakes of iodine estimated using 24-HR and FFQ

	All children		<6 mo		6–11.9 mo		≥12 mo	
	*N*	Value	*n*	Value	*n*	Value	*n*	Value
UIC								
P50 (P25 and P75)	326	145 (85, 226)	131	130 (81, 199)	114	143 (83, 235)	81	163 (113, 258)
Iodine intake, 24-h^1^								
P50 (P25 and P75)	333	83 (64, 113)	132	75 (57, 114)	117	90 (70, 113)	84	86 (67, 113)
% below AI^2^ (95% CI)	188	56 (51, 62)	79	60 (51, 68)	57	49 (39, 58)	52	62 (51, 72)
% below EAR^3^ (95% CI)	117	35 (30, 41)	63	48 (39, 57)	30	26 (18, 35)	24	29 (19, 39)
% above UL^4^ (95% CI)	2	0.6 (0.0, 2.2)	1	0.7 (0.0, 4.1)	1	0.8 (0.2, 4.6)	0	0 (0.0, 4.2)
Iodine intake, FFQ^5^								
P50 (P25 and P75)	137	101 (52, 145)	—	—	80	66 (37, 126)	57	127 (88, 164)
% below AI^2^ (95% CI)	66	48 (40, 57)			47	59 (47, 70)	19	33 (21, 47)
% below EAR^3^ (95% CI)	44	32 (24, 41)			39	49 (37, 60)	5	9 (3, 19)
% above UL^4^ (95% CI)	20	15 (9, 22)			9	11 (5, 20)	11	19 (10, 32)

Presented for all children (*N* = 333) and for 3 age group categories.

Abbreviations: UIC, urinary iodine concentration; 24-HR, 24-h dietary recall; FFQ, food frequency questionnaire; EAR, Estimated average requirement; UL, Upper intake level, AI, Adequate intake, CI, confidence interval.

1 Estimated usual iodine intakes (μg/d) calculated using the Multiple Source Method [25].

2 Adequate intake from the Nordic Nutrition Recommendations 2023 (90 μg/d for infants <12 mo of age and 100 μg/d for children ≥12 mo of age [30].

3 Estimated average requirement of 72 μg/d for children <2 y of age [32].

4 Upper intake level of 200 μg/d from the European Food Safety Authority [31].

5 FFQ data was available for 251 children, but calculations of energy and iodine intakes were only computed for children ≥6 mo of age.

**TABLE 3:** Spearman’s rank correlation coefficient between children’s UIC and children’s iodine intake, BMIC, maternal UIC and maternal iodine intake

	Children’s UIC	Children’s iodine intake^1^	BMIC	Maternal UIC	Maternal iodine intake^1^
Children’s UIC	1.00				
Children’s iodine intake^1^	0.408	1.00			
	(*P* < 0.001)				
BMIC	0.431	0.669	1.00		
	(*P* < 0.001)	(*P* < 0.001)			
Maternal UIC	0.231	0.342	0.325	1.00	
	(*P* = 0.001)	(*P* < 0.001)	(*P* < 0.001)		
Maternal iodine intake^1^	0.203	0.216	0.337	0.170	1.00
	(*P* = 0.004)	(*P* = 0.002)	(*P* < 0.001)	(*P* = 0.017)	

Abbreviations: UIC, urinary iodine concentration; 24-HR, 24-h dietary recall; BMIC; breast milk iodine concentration, MSM, Multiple Source Method.

Estimated usual iodine intakes (μg/d) calculated using 24-HRs and the MSM [25].

1 Estimated usual iodine intakes (μg/d) from Aarsland et al. [23] using 24-HRs and the MSM [25]. The median (P25 and P75) iodine intake was 151 μg/d (103 and 216) in all females (*N* = 300), including iodine from food and supplements [23].

**FIGURE 2:**
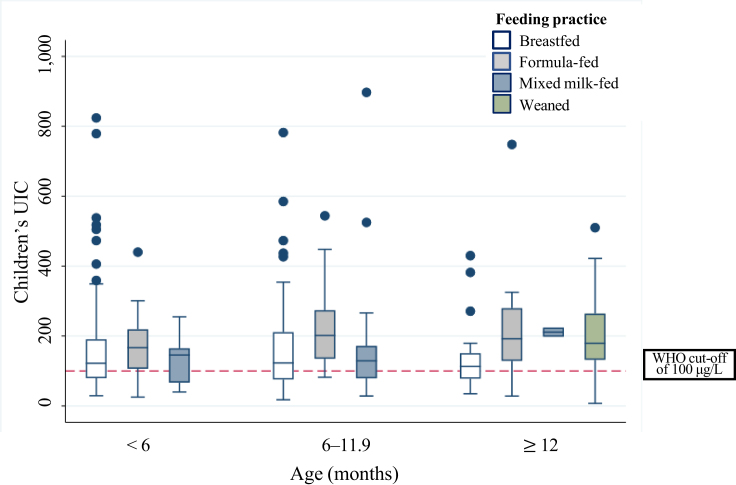
Box plot of children’s urinary iodine concentration (UIC), stratified by 3 age groups. The horizontal line in the box indicates the median UIC, the box indicates the interquartile range (IQR) (P25–P75), the whiskers represent observations within 1.5 times the IQR, and the filled circles are outliers (defined as a value >1.5 length of the box). Sample sizes in the age groups were as follows: <6 mo (*n* = 131): breastfed (*n* = 101), formula fed (*n* = 16), mixed milk-fed (*n* = 14); 6–11.9 mo (*n* = 114): breastfed (*n* = 71), formula fed (*n* = 30), mixed milk-fed (*n* = 13); ≥12 mo (*n* = 81): breastfed (*n* = 21), formula fed (*n* = 17), mixed milk-fed (*n* = 2), and weaned (*n* = 41).

The median UIC was 145 μg/L and above the current recommended cutoff of 100 μg/L in all age groups (Table 2). The lowest UIC was among the breastfed children regardless of age (Figure 2). However, within the age groups, there were no significant differences in UIC between different feeding practices, except for infants aged 6–11.9 mo, where formula-fed infants had substantially higher UIC (202 μg/L) compared with breastfed (123 μg/L) and mixed milk-fed infants (129 μg/L) (*P* = 0.0045).

For all children, the median usual iodine intake based on the 24-HRs was 83 μg/d. A total of 56% (*n* = 188) had usual iodine intakes below the AI, 35% (*n* = 117) had iodine intakes below the EAR of 72 μg/d (suboptimal intake), whereas <1% (*n* = 2) exceeded the UL of 200 μg/d (excessive intake). For infants aged 6–11.9 mo, the median iodine intake was just within the AI of 90 μg/d, whereas for the other 2 age groups (<6 and ≥12 mo), the median iodine intakes were below the AIs of 90 and 100 μg/d, respectively (Table 2). Furthermore, in the youngest 2 age groups, breastfed infants had significantly lower iodine intakes than formula-fed and mixed milk-fed infants (<6 mo: 67 μg/d compared with 119 and 109 μg/d, respectively, *P* < 0.001, and 6–11.9 mo: 82 μg/d compared with 103 and 115 μg/d, respectively, *P* < 0.001). For children ≥12 mo of age, there were no notable differences in iodine intake between different feeding practices (*P* = 0.6148). Besides breast milk and formula, the main dietary iodine sources were industrial infant porridge and cow’s milk, contributing 14.7% and 7.3% to the iodine intake, respectively (Table 4).

**TABLE 4:** Contribution (%) from different food groups to the iodine intake, based on 24-HRs from 333 children (2 × 24-HRs from 310 children)

Food group	Contribution of food group to the total iodine intake (%)			
	All children (*N* = 333)	<6 mo (*n =* 132)	6–11.9 mo (*n =* 117)	≥12 mo (*n =* 84)
Dairy products	12.6	<0.1	2.7	45.5
Milk	7.3		0.8	27.2
Yogurt, cottage cheese, and quark	3.0		0.8	10.4
White-colored cheese^1^	0.8		0.3	2.8
Whey cheese	1.4		0.7	4.6
Other dairy products^2^	0.1		<0.1	0.5
Fish and seafood	4.9	0.2	5.5	11.1
Lean/semi-fat fish and related products	2.2	0.2	4.5	2.0
Fatty fish	0.1	0.0	0.1	0.2
Other fish products	2.6	0.0	0.9	8.9
Eggs	1.2	0.1	1.1	3.2
Bread, cereals, seeds, and nuts	15.9	6.4	27.8	12.6
Industrial infant porridge	14.7	6.1	27.1	9.3
Other products	1.2	0.3	0.7	3.3
Vegetables, fruits, and berries	1.1	0.2	1.1	2.2
Poultry, meat, and meat products	0.3	<0.1	0.2	0.7
Butter, margarine, and oils	0.2	<0.1	0.2	0.6
Non-dairy beverages^3^	0.2	<0.1	0.1	0.7
Supplements	0.0	0.0	0.0	0.0
Mixed dishes and other products	1.9	<0.1	1.9	4.8
Breast milk	40.5	67.3	36.5	7.0
Formula	21.2	25.9	22.9	11.6

Presented for all children and for the 3 age group categories.

Abbreviation: 24-HR, 24-h dietary recall.

1 Solid and cream cheese.

2 Cream milk, crème fraiche, sour cream and ice cream.

3 Juice, soda and plant-based milk replacements.

Calculation of iodine intake from the FFQ was only computed for children >6 mo of age, because the FFQ used for the younger children only quantified the consumption amount of certain food items. As indicated by Table 2, 48% (*n* = 66) of the children had iodine intakes below the AI, 32% (*n* = 44) had intakes below the EAR, and 15% (*n* = 20) had intakes above the UL.

### Correlation between children’s UIC, children’s iodine intake, and maternal iodine nutrition

The correlation coefficients between children’s UIC and children’s iodine intake, BMIC, maternal UIC, and maternal iodine intake are presented in Table 3. In the lactating females who provided breast milk samples (*n* = 220), the median (P25 and P75) BMIC was 74 μg/L (48 and 110). The median (P25 and P75) UIC in all mothers (*N* = 330) was 92 μg/L (54 and 148), indicating inadequate maternal iodine status. There was a high and statistically significant correlation between children’s iodine intake and BMIC, whereas a moderate correlation was found between children’s UIC and BMIC, maternal UIC, and maternal iodine intake.

## Discussion

To our knowledge, this is the first study to present data on iodine nutrition in young children in Norway, including both UIC, estimated iodine intake, and associations with maternal iodine nutrition.

In the present study, the median UIC in all children was 145 μg/L, which indicates adequate iodine status according to the current WHO cutoff of 100 μg/L for children <2 y of age. However, this UIC cutoff for infants and toddlers has been questioned as it does not align with the WHO dietary intake recommendations for this age group. The UIC cutoff is based on goiter prevalence in school-aged children, and it does not consider that younger children have lower urine volumes compared with school-aged children [16]. The WHO is currently reviewing their cutoffs for ID, and a cutoff of a median UIC >200 μg/L has been proposed by others [16]. Using the proposed cutoff of >200 μg/L, the median UIC among the children in our study would be defined as insufficient in all age groups. In addition, the dietary assessment of the same children indicated that a large proportion had usual iodine intakes below the EAR.

The lowest median UIC was among the breastfed children in all age groups. Another recent study in Norway (*N* = 113) found lower UIC in breastfed infants compared with mixed milk-fed and weaned infants at 3 and 6 mo of age, but not at 9 mo of age [34]. Also, other studies in Norway and elsewhere have reported lower UIC among breastfed children compared with other feeding practices [6,35,36]. Of the few previous studies from Norway reporting data on UIC in children 0–2 y of age, a median UIC in the range of 82–210 μg/L has been reported [[34], [35], [36], [37], [38]]. In these Norwegian studies, the lowest levels have been reported in breastfed infants aged 3 mo (median UIC 82 μg/L) [34], and the highest levels have been reported in weaned infants ≤1 y of age (median UIC 210 μg/L and median age ∼5 mo) [35].

**FIGURE 3:**
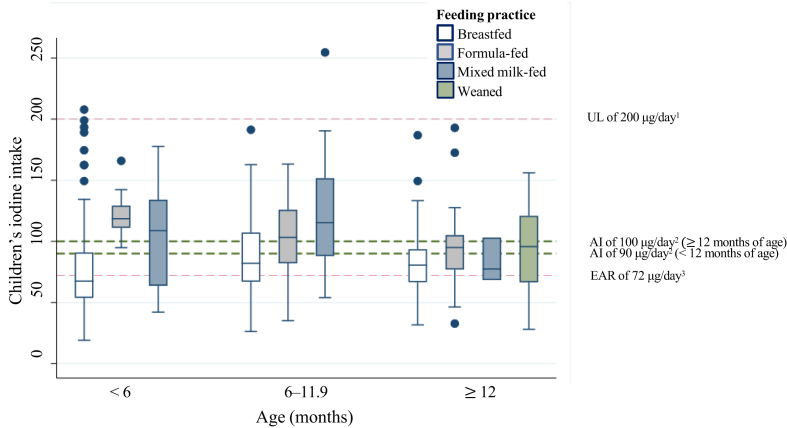
Box plot of children’s dietary iodine intake by 24-HRs, stratified by the 3 age groups. The horizontal line in the box indicates the median iodine intake, the box indicates the interquartile range (IQR) (25th–75th percentile), the whiskers represent observations within 1.5 times the IQR, and the filled circles are outliers (defined as a value >1.5 length of the box). Sample sizes in the age groups were as follows: <6 mo (*n* = 132): breastfed (*n* = 102), formula fed (*n* = 16), mixed milk-fed (*n* = 14); 6–11.9 mo (*n* = 117): breastfed (*n* = 72), formula fed (*n* = 32), mixed milk-fed (*n* = 13); ≥12 mo (*n* = 84): breastfed (*n* = 22), formula fed (*n* = 17), mixed milk-fed (*n* = 3), and weaned (*n* = 42). 24-HR, 24-h dietary recall. ^1^Upper intake level of 200 μg/d from the European Food Safety Authority [31]. ^2^Adequate intake from the Nordic Nutrition Recommendations 2023 (90 μg/d for infants <12 mo of age and 100 μg/d for children ≥12 mo of age [30]. ^3^Estimated average requirement of 72 μg/d for children <2 y of age [32].

On the basis of the repeated 24-HRs in this study, which was the main method chosen for dietary assessment, 35% of the children had suboptimal usual iodine intakes (below the proposed EAR of 72 μg/d). Similar to the UIC, the iodine intake differed between age groups and feeding practice (Figure 3), with the highest proportion of children with suboptimal iodine intakes among breastfed children in the youngest age group (59 out of 102; 58%). The iodine intake of exclusively breastfed infants is largely determined by the BMIC. For instance, with a BMIC equal to the median BMIC of 74 μg/L in this study, a 1-mo-old fully breastfed infant will be provided with 46 μg iodine/d (74 μg/L × 0.624 L), which is far below the EAR of 72 μg/d. Conversely, a fully formula-fed infant receiving the same daily volume of 0.624 L will receive 87 μg/d, assuming an iodine concentration in formula equal to the median iodine concentration of the formulas in this study of 140 μg/L. There is currently no established BMIC cutoff to categorize iodine sufficiency in lactating females, but to maintain positive iodine balance in infants, a BMIC of 100–200 μg/L has been suggested [16]. The median BMIC of 74 μg/L in this study falls short of this proposed range and suggests that infants having breast milk as their primary food source are likely to have suboptimal intakes of iodine. In our study, both children’s UIC and iodine intake were positively associated with all markers of maternal iodine nutrition (BMIC, UIC, and estimated iodine intake). This supports the importance of maternal iodine nutrition in providing sufficient iodine intake in young children and is also consistent with the findings of other studies [6,[39], [40], [41]].

The finding that 35% of the children in this study had suboptimal usual iodine intakes (below the EAR) is highly concerning and suggests that young children in Norway are at risk of ID. Although assessment of iodine intakes in populations should be based on the EAR [32], few studies in this age group have reported such data. The Little in Norway Study from 2018 reported that none of the 18-mo-old children (*N* = 416) had suboptimal iodine intakes [37]. However, the authors used a lower EAR than in the current study (65 μg/d from the Institute of Medicine), included a higher age group, and breastfed children were excluded from the iodine intake estimations because of limited knowledge of consumption volumes. Thus, our study is the first of its kind to reveal such concerning levels of iodine intakes across different child age groups and feeding practices.

The lack of a national salt iodization strategy in Norway has been largely attributed to concerns over the potential risks of excessive iodine intakes in young children, specifically 1- and 2-y-olds [42]. Children of this age have been considered vulnerable groups to iodine excess as they have immature thyroid glands and are less capable of adapting to high iodine intakes compared with adults and older children [43]. Children with a high consumption of cow’s milk are at higher risk of excess iodine. Thus, with an increase in the iodine concentration in salt (from 5 to 20 μg/g; in line with the recent law change [20]), the National Council of Nutrition has stressed the importance of limiting milk and yogurt intake to 500–600 mL/d for young children [44]. In our study, the mean (SD) intake of cow’s milk (including yogurt) among consumers (*n* = 96) was 260 mL/d (190). Moreover, our data showed that only 2 children (<1%) had usual iodine intakes above the UL (200 μg/d), suggesting that low iodine intakes were a greater concern than excessive intakes in this population.

A considerable strength of the current study was the use of both UIC and extensive dietary data collected from a large sample of children from randomly selected municipalities. Furthermore, it was a strength that we had 2 24-HRs from almost all children, and that we used the MSM to adjust for within-subject variability and estimate usual iodine intakes. A limitation is that there was an oversampling of children below 1 y of age compared with children above 1 y of age, likely because of the higher frequency of consultations at the public health care center during the first year of life compared with the subsequent years. Also, many of the children spent part of the day away from home or with another caregiver, leading to varying levels of knowledge and awareness among the mothers regarding their child**’**s diet. Dietary assessment in young children is already a complex task that faces several respondent and observer considerations related to portion sizes, food spillage, and consumption of breast milk and/or formula. Another limitation is that we used spot urine samples to assess UIC, which does not reflect the usual iodine status. However, our results rely predominantly on the estimated intakes of iodine derived from repeated 24-HRs. Finally, we did not measure any biomarkers of thyroid function, which is warranted to provide a better picture of iodine nutrition and potential health consequences in this age group.

In conclusion, this study suggests that the iodine status of children ≤2 y of age in Norway is adequate on a population level, as indicated by a median UIC >100 μg/L in the overall group. However, the UIC cutoff is debated, and the extensive dietary data indicate that more than a third of the children have suboptimal usual iodine intakes as shown by intakes below the EAR. Our results suggest that many children will benefit from iodine fortification, both directly through increased iodine from solid foods and indirectly through increased iodine in breast milk. Moreover, our results suggest that risk of iodine excess in young children is low.

## Acknowledgments

We are thankful to the mothers and children who participated in this study, and to the healthcare staff who provided invaluable assistance during the recruitment process and data collection.

## Author contributions

The authors’ responsibilities were as follows—TAS, KSB, BSS, SK: designed the research; TAS, KSB: coordinated large parts of the data collection; KSB, BSS, SK, TEA: took part in recruitment; KRN, ITF, TEA: prepared the urine and breast milk samples before analyses; THØ, SL, ELFG: iodine analyses of urine and breast milk samples; TEA, SNS, BSS, KSB, SK, TAS: analyzed the data and performed the statistics; TEA: wrote the first draft of the paper; TAS, KSB, SNS: supervised; and all authors: read, contributed to and approved the final manuscript.

## Conflict of interest

The authors report no conflicts of interest.

## Funding

This work was supported by the Innlandet Hospital Trusts research fund (150407).

## Data availability

Requests for data presented in this study can be made to the authors. To meet ethical requirements for the use of confidential data, requests must be approved by the Regional Committee for Medical and Health Research Ethics in Norway.
